# The Arcana of Zinc

**DOI:** 10.1016/j.tjnut.2025.01.004

**Published:** 2025-01-08

**Authors:** Wolfgang Maret

**Affiliations:** Department of Nutritional Sciences, School of Life Course and Population Sciences, Faculty of Life Sciences and Medicine, King’s College London, London, United Kingdom

**Keywords:** Zinc, zinc proteome, micronutrients, biomarkers, metallostasis

## Abstract

This perspective discusses the essential micronutrient zinc, which functions in >3000 human proteins (the zinc proteome), and the implications of three aspects to ascertain an adequate zinc status for human health. First, the advent of highly sensitive fluorescent (bio)chemicals revealed cellular pools of zinc ions involved in signaling and secretion from cells for paracrine, autocrine, and possibly endocrine functions. Zinc signaling adds a yet unaccounted number of targeted proteins to the already impressive number of zinc proteins. Second, cellular zinc concentrations are remarkably high in the order of the concentrations of major metabolites and, therefore, at the cellular level zinc is not a trace element. Zinc is also not an antioxidant because zinc ions are redox-inactive in biology. However, zinc can express indirect pro-oxidant or proantioxidant effects depending on how cellular zinc is buffered. Zinc sites in proteins and other biomolecules can become redox-active when zinc is bound to the redox-active sulfur donor atom of cysteine. This interaction links zinc and redox metabolism, confers mobility on tightly bound zinc, and has implications for treating zinc deficiency. Third, the concept of zinc deficiency in blood as the only measure of an inadequate zinc status needs to be extended to zinc dyshomeostasis in cells because overwhelming the mechanisms controlling cellular zinc homeostasis can result in either not enough or too much available zinc. We need additional biomarkers of zinc status that determine cell-specific changes and perturbations of the system regulating cellular zinc, including functional deficits, and address the multiple genetic and environmental factors that can cause a conditioned zinc deficiency or overload. Considering the wider context of altered zinc availability in different organs, cells, and organelles impinges on whether zinc supplementation will be efficacious and adds another dimension to the already high health burden of zinc deficiency and its sequelae worldwide.

## Introduction

Zinc, i.e., the zinc(II) ion, is an essential micronutrient for the growth and development of all forms of life [1]. Essentiality of zinc was established for a fungus 155 y ago, for rats 90 y ago, and for humans 60 y ago [[2], [3], [4]]. Given this long history, it is surprising that the biological roles of zinc have not been exploited to the extent necessary for preventing and treating human diseases. In this perspective, therefore, I shall discuss reasons why it was difficult to recognize the full significance of this elusive micronutrient [5], draw attention to concepts that need to be examined, and identify research gaps that would allow us to explore new directions in nutrition research and practice and biomarker research with wide-ranging consequences for improving individual and public health. The need for this assessment arises because recent developments allow estimates of the number of zinc-dependent functions, add another layer of complexity to the interactions of zinc with proteins, and provide a basis for defining future functional biomarkers of cellular zinc status.

## The Development of Zinc Biology and Biochemistry

**FIGURE 1:**
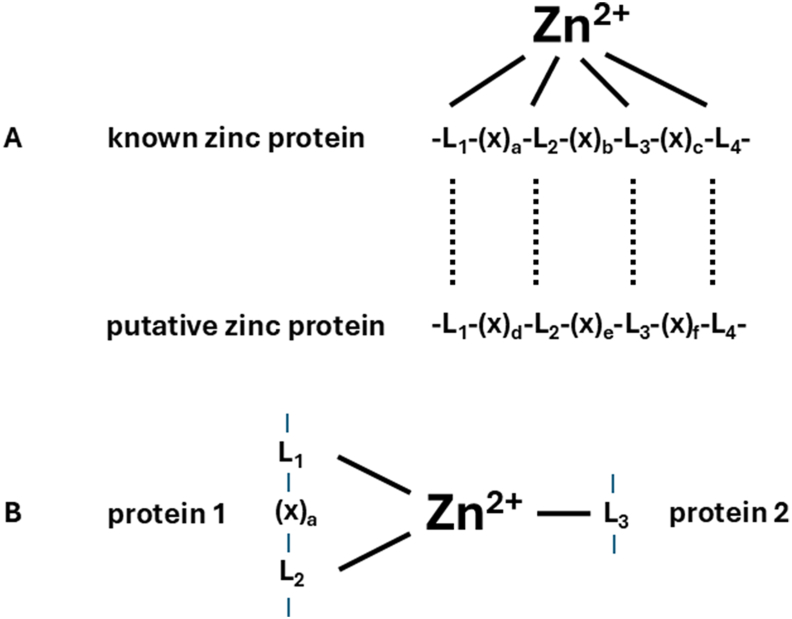
Zinc signatures in proteins. (A). Prediction of zinc sites in proteins. Zinc-binding sites of zinc proteins with known zinc ligands (L)—usually 3 for catalytic zinc sites and 4 for structural zinc sites from the side chains of histidine, cysteine, glutamate, or aspartate—can be used to search for analogous sites in proteins for which zinc-binding sites have not been established. The number of amino acids (X) between the ligands can vary (a-f). (B). Zinc binding at protein interfaces mediating protein-protein interactions. One or two zinc-binding ligands in a protein do not give sufficient information for a predictive signature. Nevertheless, zinc binding can occur when additional ligands stem from a different protein.

Starting with the discovery of zinc in bovine carbonic anhydrase in 1939, the field of zinc biochemistry grew with finding of zinc as a catalytic cofactor in hundreds of enzymes [6,7]. The discovery of zinc finger proteins established zinc as a frequent structural cofactor in proteins [8,9]. With the advent of many 3D structures of zinc proteins becoming available, it was recognized that zinc proteins have characteristic signatures (“motifs”) for zinc binding in their amino acid sequences. These signatures have several amino acids between the amino acids that provide the ligand donor atoms for zinc and allow the prediction of zinc-binding sites in proteins that are not known to be zinc proteins [10] (Figure 1A). When sequences of entire genomes became available, they were mined for such signatures. This led to the remarkable estimate that >3200 human proteins are zinc proteins [11], i.e., ≥10% of all human proteins employ zinc as a cofactor. The recognition of signatures fails, however, if the zinc-binding ligands stem from different proteins (Figure 1B). There is a growing appreciation for such zinc binding at the interfaces between different proteins [12], increasing the number of zinc-protein interactions. Thus, zinc’s plethora of functions makes zinc indeed a key element of life and led to the notion that zinc galvanizes biology [13,14].

## Zinc Ion Signaling

In 1960, Nobel laureate Milstein [15] reported that zinc inhibits the magnesium enzyme phosphoglucomutase with picomolar affinity. He concluded that zinc ion concentrations in muscle must be in the low picomolar range in order not to inhibit the enzyme. However, the significance of the finding, namely that zinc ions serve as physiological regulators at such low concentrations and that zinc interferes with magnesium sites in proteins, became apparent only 40 y later when a role of zinc ions (Zn^2+^) in intracellular and extracellular communication was identified. The discoveries became possible with the advent of fluorescent (bio)chemicals that allow the tracing of zinc ions in cells at their very low concentrations. Thus, an extracellular stimulus was found to generate an intracellular so-called zinc wave from the endoplasmic reticulum [16]. The released zinc ions serve as second messengers for modulating signal transduction pathways. One way of releasing zinc ions includes the phosphorylation of the zinc ion channel Zip7 in the endoplasmic reticulum and ensuing secretion of zinc ions into the cytoplasm [17]. These discoveries were preceded by establishing the role of zinc ions in extracellular communication. Zinc ions are secreted as neurotransmitters from presynaptic vesicles in a specific set of neurons [18]. Subsequent work showed that exocytosis of zinc ions stored in cellular vesicles occurs in many other cells as a way of signaling between cells [19,20]. The insulin-storing vesicles of pancreatic ß-cells are the best-known example. Zinc ions are secreted together with insulin and have an autocrine effect on ß-cells, a paracrine effect on α-cells, modulating glucagon secretion, and possibly an endocrine effect on the liver, affecting insulin clearance [21,22]. Another remarkable example of the secretion of zinc ions is the zinc sparks observed in fertilized mammalian oocytes [23]. Meanwhile, physiological concentrations of fluctuating zinc ions have been shown to inhibit many proteins in signaling pathways, notwithstanding that the overall effect of the inhibition can be the activation of a pathway. For example, picomolar to low nanomolar concentrations of zinc ions inhibit protein tyrosine phosphatases, including PTP1B, the major phosphatase regulating the insulin receptor [24,25]. Inhibiting this phosphatase keeps the insulin receptor, a tyrosine kinase, in its phosphorylated, active state for insulin signaling. In this way, zinc ions can modulate a wide range of cellular signaling pathways relying on phosphorylations. The exact number of targeted proteins in zinc ion signaling is not known [26]. It is certainly not insignificant and these regulatory sites in transient interactions with proteins should also be included in the already impressive number of a few thousand human zinc proteins in the zinc proteome, i.e., all the proteins with a function depending on zinc. The sheer number of functions of zinc as a cofactor in thousands of proteins gives zinc much prominence among the nutritionally essential microminerals. To put this importance into perspective, a comparison with vitamins as micronutrients is useful because, for some of them, the counts of cofactor-dependent enzymes are 1 to 3 orders of magnitude less than for zinc. For example, in humans, vitamin B12 is required for only two enzymes, methylmalonyl coenzyme A mutase and methionine synthase, whereas vitamin K is involved in 19 reactions [27]. Even for pyridoxal phosphate, the active form of vitamin B6, only 56 human genes code for pyridoxal phosphate-dependent enzymes [28].

## Control of cellular zinc homeostasis and the concept of zinc dyshomeostasis

For zinc ions to serve as signaling ions, the system of cellular homeostatic control requires additional molecular mechanisms to allow fluctuations of zinc ions. The system turns out to be remarkably complex and intricate.

At least 40 proteins control cellular zinc homeostasis, also referred to as zinc metallostasis, and coordinate the numerous functions of zinc. They include two dozen proteins of the Zip (SLC39A1-14) and ZnT (SLC30A1-10) families of zinc transporters, a dozen metallothioneins, and minimally one zinc sensor (metal regulatory element binding transcription factor 1, MTF-1) controlling zinc-dependent gene expression, and one zinc chaperone (Zn-regulated GTPase metalloprotein activator, ZNG1) [[29], [30], [31], [32]]. The proteins controlling zinc are regulated in cell-specific ways, interact with multiple proteins, and are integrated into cellular metabolism and signaling. Zinc transporters determine cellular influx and efflux of zinc ions as well as their cellular distribution into and from cellular organelles. Any perturbation of zinc transport by this large set of zinc transporters and resulting changes in zinc fluxes is an important factor for developing diseases [33,34]. Accordingly, these zinc homeostatic proteins are becoming important targets for treating diseases. The number of proteins involved in the control of just this single micronutrient is another manifestation of zinc’s inimitable role. The system of zinc metallostasis emphasizes the many factors that can affect the cellular availability of zinc.

Perhaps the most critical piece of missing information is whether a hierarchy exists in the cellular distribution and allocation of zinc to proteins. Is zinc binding in zinc enzymes more important than binding to zinc transcription factors, to structural zinc sites, at the interface of proteins, or the role of zinc in zinc ion signaling? Or is zinc allocation even prioritized within each of these functional groups? If zinc becomes limiting, are the thousands of zinc proteins all affected to the same extent, or is preference given to maintaining the functions of only some of them? Given the range of affinities for zinc binding to proteins, one expects that zinc proteins are affected differentially. This difference in affinities offers the possibility to categorize zinc-protein biomarkers for zinc status because the proteins with lower affinity for zinc are expected to be affected by zinc deficiency first, whereas the ones with higher affinity would gradually lose their zinc when zinc deficiency is more severe and/or lasts longer. The recent finding of a genuine zinc chaperone protein supplying zinc to methionine aminopeptidase 1 would suggest that at least one process, namely the removal of the N-terminal methionine of newly synthesized proteins, is prioritized [35]. Methionine aminopeptidase 1 is part of the nascent polypeptide-associated complex (NAC) on the ribosome. NAC is essential for cotranslational protein processing in higher eukaryotic organisms [36]. Another process of proteostasis also appears to require a chaperone protein to channel zinc from ZnT transporters to client proteins in the secretory pathway [37]. Clarifying whether a hierarchy in cellular zinc distribution and function exists is paramount for defining biomarkers of zinc status.

Knowing the correct physiological zinc concentrations in cells and when they are compromised requires information about how cellular zinc metallostasis is controlled and how cellular zinc ions are redistributed in a spatiotemporal way for their different functions. Here, two important concepts are to be reckoned with: zinc buffering and the interaction between zinc metabolism and redox metabolism.

## Cellular Zinc Buffering and Redox Control of the Pool of Available Zinc Ions

In as much as physiological pH values are critical for living organisms, maintaining physiological pMe values for metal ions, pZn in the case of zinc, is equally important. The formalism to describe zinc buffering is analogous to that describing proton buffering except that the zinc dissociation constant (p*K*_Zn_) and the ratio of the concentrations of free ligand (L) to zinc-bound ligand (ZnL) are used instead of the acid dissociation constant (p*K*_a_) and the ratio of base (B) to acid (A) (Equations 1 and 2).

Equation 1 $$\text{pH}=pK_{a}+\log\left(B/A\right)$$

Equation 2 $$\text{pZn}=pK_{\text{Zn}}+\log\left(L/\text{ZnL}\right)$$

The pZn defines the concentration of zinc ions available from the total zinc concentration. The concept of metal buffering is critically important for physiology as every essential metal ion needs to be buffered in a certain range in order not to interfere with the functions of other metal ions [38]. Thus, Zn^2+^ binds more tightly to proteins than Mg^2+^ and the paradigmatic signaling ion Ca^2+^. Because of this higher affinity, Zn^2+^ is buffered differently, and the concentrations of nonprotein bound zinc ions are in the picomolar to low nanomolar range and thus lower than those of calcium ions [39]. It allows Zn^2+^ signaling to complement Ca^2+^ signaling by reaching different cellular targets [19]. Also, the two ions have opposite cellular gradients. Whereas calcium has higher concentrations outside cells, zinc has higher concentrations inside cells. This cellular acquisition of zinc requires discussion of an important concept. Referring to zinc as a trace element employs a term that was introduced at a time when it was impossible to measure accurately the low concentrations of some metal ions. The term refers to substances that occur at concentrations less than 0.1% or 1000 parts per million. The total cellular concentrations of zinc are hundreds of micromolar and thus as high as those of major metabolites such as glucose-6 phosphate or citrate [40]. One would hardly consider such concentrations a trace in the general discussion of biochemistry. Therefore, in the context of all its cellular functions, the term trace element distracts from the real importance of zinc. Among all the proteins and low molecular compounds that contribute to cellular zinc buffering, metallothioneins have a major function as a dynamic zinc buffer and in storing zinc temporarily [41]. Metallothioneins can adjust the two parameters that determine zinc buffering: the total capacity to bind zinc and the pZn values that determine the zinc ion concentration that is available from the total zinc concentration. In other words, metallothioneins determine the size and concentrations of a pool of available zinc ions. The pool is functionally critically important for signaling, gene expression, and protein regulation and it is cell-specific due to the different expression profiles of metallothioneins. Furthermore, the availability of zinc from metallothioneins depends on prevailing pH values and the redox state, both of which differ in the various cellular organelles. In as much as deviation from a physiological pH value spells disaster for cells, so does deviation from a physiological pZn value. It can mean that either too much or not enough zinc is available. Defining zinc buffering, therefore, is critical for zinc metallostasis and distinguishing essential from toxic actions of zinc [42].

Zinc is redox-inactive in biology, always remaining Zn^2+^. However, a fundamental principle is concealed in the interaction of zinc with the sulfur donor atom of cysteine in many zinc proteins [43]. Because the sulfur in cysteine is redox-active, the interaction confers redox activity on zinc coordination environments with cysteine(s) and links zinc and redox biology in the following way. Oxidation of the sulfur donor atom of cysteine reduces the zinc-binding capacity and makes more zinc ions available in the cell (lowering the pZn), whereas reduction of oxidized cysteine sulfur restores zinc binding and makes fewer zinc ions available (raising the pZn) [44]. Metallothioneins, with their zinc coordination exclusively to cysteine sulfur, therefore, are redox-controlled zinc buffers [41]. Under physiological oxidative stress, which is referred to as oxidative eustress [45], this molecular principle is a means of regulating cellular zinc via redox signaling, whereas under pathological oxidative stress, which is referred to as oxidative distress [45], too much zinc is made available. These concepts of buffering and redox control of zinc have wide-reaching consequences for the nutritional role of zinc and its relation to oxidants, antioxidants, and zinc-binding molecules.

## The Quest for Biomarkers of Cellular Zinc Status and the Specific Indications for Zinc Supplementation

Remarkably, zinc biomarker research and zinc supplementation has been pursued for several decades. Thus, already in 1990, it was suggested that additional zinc-binding proteins in blood plasma could be employed for assessing zinc status [46]. Exemplary editorials for the “unheralded” nutrient zinc [47] and the “neglected problem” of human zinc deficiency [48] were published >25 y ago, and the subject matter was treated in comprehensive articles that appeared in regular intervals thereafter [49]. A review and meta-analysis of biomarkers for human zinc status covering the period 2007–2022 includes 48 putative biomarkers in addition to the 32 biomarkers previously evaluated in the 2009 European Micronutrient Recommendations Aligned project [50,51]. Plasma/serum and urinary zinc responded to zinc intake in healthy participants when zinc was given as zinc sulfate, zinc acetate, zinc gluconate, or zinc methionine [50]. Cut-offs defining zinc deficiency clinically are widely accepted only for plasma/serum zinc, albeit with the caveat of confounders such as inflammation when zinc is withdrawn from blood in the acute phase response and redistributed to tissues. Also, further evidence is needed for the usefulness of plasma/serum zinc determinations in pregnant, lactating, and postmenopausal women [50]. At present, blood plasma/serum zinc remains the only biomarker of zinc status in clinical practice. It represents only 0.1% of total zinc in the human body and is a component of the exchangeable zinc pool, which itself was classified as “unclear” as an effective biomarker [50]. The authors conclude that although there is no scarcity of putative biomarkers, none of the additional biomarkers can presently distinguish between inadequate and optimal zinc status, and therefore, more investigations are needed to establish reliability and sensitivity [50]. They also suggest that ≥3 biomarkers would provide a better measure of zinc status and the efficacy of additional zinc intake. A zinc status index could include specific functional biomarkers that are responsive to the availability of zinc, such as the composition of the gut microbiome, ratios of specific fatty acids in erythrocytes, and the expression of specific genes in tissues [52]. Importantly, the reviewed and analyzed investigations were performed in healthy individuals [50]. In clinical practice, however, we want to know the response to zinc intake in patients and define cases of subclinical zinc deficiency linked to various adverse health outcomes.

Plasma/serum zinc as a single biomarker of zinc status is clearly insufficient. It can also be inconclusive for at least two reasons. First, it is mainly utilized as a sign of zinc deficiency. Zinc dyshomeostasis, however, can manifest in either too much or not enough cellular zinc ions being available, or it may reflect a functional deficiency, in which zinc is unavailable from an intracellular organelle or from proteins such as metallothioneins. All the additional zinc biomarkers were also explored experimentally on the premise that they detect zinc deficiency as the only sign of an abnormal zinc status [45,50,51]. Therefore, we need to examine a key aspect underlying our assumptions, namely whether a systemic zinc deficiency in blood necessarily assesses a cellular zinc deficiency and indicates supplementation with zinc. Second, zinc in blood does not reflect the situation in some tissues. For example, the zinc exchange rate for muscle, which contains ∼60% of body zinc, is 210 d, and thus, zinc in muscle is not in rapid equilibrium with zinc in blood [53]. With cellular zinc being involved in so many processes and underlying such a complex control, which can be compromised by so many factors, the starting point in searching for and evaluating additional biomarkers of zinc status is an understanding of how cellular zinc is buffered, regulated, and distributed to the many proteins that require it for numerous biochemical functions. At least three factors should be considered when biomarkers detect a zinc cellular dyshomeostasis and whether zinc supplementation will be efficacious: the cellular redox state, the thresholds of cellular zinc buffering, and mutations in the proteins controlling cellular zinc homeostasis.

### The cellular redox state

Zinc deficiency and zinc overload are both pro-oxidant conditions that can elicit oxidative distress. Therefore, zinc is not simply a widely propagated antioxidant, an issue that was already discussed in editorials many years ago [54,55]. Whether zinc has indirect pro-oxidant or proantioxidant effects depends on its cellular availability and the cellular redox state. Oxidative distress can initiate a cascade of events resulting in a vicious cycle in which zinc ions inhibit redox enzymes involved in the antioxidant response and thus augment oxidative distress. When too much zinc is available, it binds to additional cellular targets. Supplementing zinc then will exacerbate the condition as zinc will bind to even more unphysiological targets with adverse effects. Therefore, before considering zinc supplementation, the absence of oxidative distress should be ascertained. According to this view, it may not always be possible to restore cellular zinc homeostasis with supplemental zinc without restoring redox homeostasis with supplemental antioxidants, e.g., in diseases accompanied by oxidative distress.

### The thresholds of cellular zinc buffering

The physiological thresholds of available cellular zinc ion concentrations as determined by zinc buffering are critical for addressing what has been referred to as the zinc paradox in several other instances aside from zinc’s role as either a pro-oxidant or proantioxidant, namely that zinc can be either cytoprotective or cytotoxic, proapoptotic or antiapoptotic, or affect inflammation in opposite ways. Zinc supplementation cannot have the expected protective outcomes if too much cellular zinc is already available.

An additional zinc paradox that resulted in conflicting observations is that zinc’s effects can be cell- and tissue-specific due to differences in zinc buffering, proteomes, and metabolomes. Tissue-specific differences in the control of cellular zinc and zinc metabolism are evident from the differential expression of zinc transporters, metallothioneins, and other proteins. Therefore, biomarkers are needed for detecting zinc dyshomeostasis in specific cells and tissues. In practice, cellular zinc buffering can be measured with fluorescent zinc probes [39].

### Mutations in the proteins controlling cellular zinc homeostasis

Another important consideration is that the dozens of proteins controlling cellular zinc metallostasis have a significant number of mutations that are associated with diseases and that some of these mutations are not rare [56]. These inborn errors of zinc metabolism resulting in zinc dyshomeostasis also need to be investigated clinically. For instance, a genetic condition of *hyperz*incemia due to increased amounts of the zinc-binding protein calprotectin in blood has been reported [57]. Causes for systemic *hypo*zincemia are mutations in the intestinal zinc uptake transporter Zip4. They can result in life-threatening zinc deficiency (acrodermatitis enteropathica). But they can also be less severe. For example, mutations in Zip4 have been associated with zinc restriction as a way of protecting against parasitic disease in African populations, a case of nutritional immunity [58]. The prevalence of the more common mutations of all 24 zinc transporters in populations needs to be known for interpreting zinc analyses in blood and before applying one-for-all recommendations for daily zinc intake. We need to know how many people need different amounts of zinc in their diet, and this includes additional recommendations in the life course, in particular for the elderly that develop oxidative distress and zinc deficiency as part of the process of inflammaging, the increase of inflammatory markers as people age.

Last but not least, the many other mitigating factors that can precipitate a secondary, conditioned zinc deficiency should be considered when zinc supplementation alone is ineffective. One example is the antinutrient phytate that binds zinc in our diet and competes with zinc uptake [59].

The search for biomarkers needs to focus on zinc’s cellular functions. The additional biomarkers already explored require further attention, in particular the ones that have been linked to glucose and fatty acid metabolism and related disease states. If a hierarchy exists in zinc distribution and allocation, the challenge will be to find and integrate several biomarkers that reflect different cellular functions and the effects of different degrees of zinc deficiency. Admittedly, it is a daunting task and a difficult needle (zinc) in the haystack (zinc proteome) search that requires further guidance from zinc biology and biochemistry in terms of the affinity of zinc for proteins. Importantly, functional biomarkers of zinc status should include biomarkers of oxidative distress and other surrogates that are conditioning factors for causing zinc dyshomeostasis. Given the implications for diagnosing and treating diseases, research on functional biomarkers of zinc status should be a top priority. Functional biomarkers may correlate positively or negatively with plasma/serum zinc, or they may not correlate at all and yet indicate a perturbation of cellular zinc homeostasis. How narrow the window for zinc supplementation is in time and dosage should be established before critical risks occur for adverse effects, including interference with the metabolism of other microminerals. Treatment should include additional nutritional support if zinc supplementation alone is ineffective [60].

In summary, after several decades of scientific inquiries and observations, blood zinc remains the only biomarker of zinc status in clinical practice. The fact that blood samples are relatively easy to acquire for analysis should not limit our diagnostic tools. For example, portable X-ray fluorescence spectrometers have been employed to measure zinc in nails and hairs [61,62]. We have many potential biomarkers, but we need to put much more effort into explaining which functional deficits they identify. We need to rethink the premise of looking only for zinc deficiency as the cause of adverse effects and instead turn our attention to biomarkers of cellular zinc dyshomeostasis based on the present knowledge of the control and the multitude of biochemical functions of zinc. Testing this paradigm shift in the way we think about biomarkers of zinc status would seem to have great potential benefits for health.

## Outlook

Zinc deficiency is “perhaps the most prevalent and least understood worldwide” micronutrient deficiency and, thus, a considerable global health burden [63], and yet there is consensus that specific and sensitive functional biomarkers of zinc status are still lacking [50,[64], [65], [66]]. If one considers all the other factors eliciting zinc dyshomeostasis in cells and tissues, physiological signs of zinc deficiency alone just seem to be the tip of the iceberg, and the issues of adequate zinc status and recommended intake and possibly supplementation become a much larger concern for health. In addition to primary (nutritional) zinc deficiency, secondary conditioned zinc deficiencies are a consequence of a host of diseases such as malabsorption syndrome, liver disease, chronic renal disease, or sickle cell disease [67]. The role of zinc in immunity teaches us that zinc-deficient individuals are immunocompromised and how important zinc is for infectious and chronic diseases [68]. Proper zinc nutriture also gains importance in the context of food insecurity and several trends impinging on the quality of food. Interconnected social and economic issues, sustainability, climate change, a trend toward vegetarian diets that have less available zinc on average, consumption of ultraprocessed food, and changes in some crops that have less zinc now than in the past all make micronutrient deficiencies a “hidden hunger” not only in the developing but also in developed parts of the world. Finally, because of new industrial manufacturing practices, we are increasingly exposed to contaminants acting indirectly on zinc metabolism and to metal ions with toxic actions acting directly on zinc metabolism. One example is cadmium, which has a very long biological half-life and accumulates in the body during our life span. Availability of sufficient zinc protects against this adverse effect and against many other toxicants.

A recent editorial in this journal entitled “Has Zinc Lost its Shine?” concludes that a more holistic approach is necessary because the benefits of zinc supplementation for pneumonia and diarrhea in children are inconclusive [69]. Thus, we need to change the way we think about zinc deficiency as the only perturbation of zinc metabolism and consider other micronutrients and factors that limit the effective metabolic use of zinc. Public health in the life course, treatment of diseases, and precision nutrition all have much to gain from a yet to be developed set of robust diagnostic biomarkers that reflect the diversity of zinc’s multiple cellular functions.

## Author contribution

The sole author was responsible for all aspects of this manuscript.

## Funding

The author reported no funding received for this study.

## Conflict of interest

The author reports no conflicts of interest.
